# Evaluation of the efficacy and safety of fire needle compared to filiform needle on knee osteoarthritis: study protocol for a randomized controlled trial

**DOI:** 10.1186/s13063-020-04827-9

**Published:** 2020-11-04

**Authors:** Yuanjie Gao, Lu Liu, Bin Li, Jing Guo, Huilin Liu, Shaosong Wang, Fan Zhang, Xu Ji, Yuanbo Fu, Yizhan Wang, Jingqing Sun, Fang Yuan

**Affiliations:** 1 Acupuncture and Moxibustion Department, Beijing Hospital of Traditional Chinese Medicine, Capital Medical University, Beijing Key Laboratory of Acupuncture Neuromodulation, Beijing, China; 2 Institute of Acupuncture and Moxibustion, China Academy of Chinese Medical Sciences, Beijing, China

## Abstract

**Background:**

Knee osteoarthritis is a common clinical chronic degenerative disease associated with high morbidity and long-term disability. Previous studies have confirmed the efficacy of acupuncture on knee osteoarthritis. Fire needle acupuncture is a combination of heat and acupuncture, which may be more effective than the commonly used filiform needle acupuncture. This study is designed as a randomized controlled trial to evaluate the efficacy and safety of fire needle acupuncture compared to filiform needle acupuncture in knee osteoarthritis patients.

**Methods and analysis:**

This is a prospective randomized controlled superiority clinical trial to evaluate the efficacy and safety of fire needle acupuncture compared to filiform needle acupuncture for knee osteoarthritis. A total of 100 participants will be randomly assigned to two different groups. Participants will receive fire needle acupuncture treatment in the fire needle group, while participants in the filiform needle group will be treated with a filiform needle at the same acupuncture points as the fire needle group. All participants will receive 6 weeks of treatment (2 times per week). The primary outcome is the change of the Western Ontario and McMaster Universities Osteoarthritis Index, and the secondary outcomes include the change of the visual analog scale and 12-item Short Form Health Survey from baseline to endpoint.

**Ethics and dissemination:**

Ethical approval of this study was granted by the Research Ethical Committee of Beijing Hospital of Traditional Chinese Medicine Affiliated to Capital Medical University (2018SB-066). Written informed consent will be obtained from all participants. Outcomes of the trial will be disseminated through peer-reviewed publications.

**Trial registration:**

Chinese Clinical Trial Registry ChiCTR1800019579. Registered on November 18, 2018

## Background

Osteoarthritis [1] is a chronic degenerative disease, which is caused by the imbalance between synthesis and degradation of articular chondrocytes, extracellular matrix, and subchondral bone tissues, under the combined action of biological and mechanical factors. Characterized by joint pain, tenderness, stiffness, joint swelling, limited mobility, and joint deformity, osteoarthritis is a common disease in middle-aged and elderly people, with an overall prevalence of primary osteoarthritis in people over 40 years old of 46.3% and in people over 70 years old of 62.1%. Increasing with age [2], it consumes a large amount of financial and medical resources and has become a serious social burden. A previous study showed that the most commonly affected joint in osteoarthritis is the knee joint [3].

The treatments for osteoarthritis include education, physical therapy, surgical intervention (minimally invasive surgery and conventional surgery), and pharmacological treatment. The pharmacological treatment mainly includes fast-acting medicines, such as non-steroidal anti-inflammatory analgesics, and slow-acting medicine, such as glucosamine sulfate and pentosan sodium polysulfate. In addition, sodium hyaluronate and hormones can be injected into the joint cavity to relieve symptoms. However, the effect of the pharmacological treatments is relatively short, and there are potential adverse events [4, 5]. Adverse events reported include minor gastrointestinal side effects by glucosamine [6–8], renal side effects by non-steroidal anti-inflammatory drugs [9], and cartilage volume loss by intra-articular (IA) corticosteroids [10].

Acupuncture therapy is a simple, inexpensive, and minimally invasive treatment, which has been applied in clinical practice in more than 160 countries and has become the mainstream of complementary and alternative medicine all over the world. Traced back to the Han Dynasty, it was already recorded in *Huangdi Neijing* that acupuncture could treat osteoarthritis, and the efficacy has been confirmed by numerous modern clinical trials and mechanism researches [3, 11, 12]. It is reported in the Cochrane systematic review that acupuncture has clinically relevant short-term improvements in osteoarthritis pain and function, though the benefits are small, and do not meet the pre-defined thresholds for clinical relevance [13]. However, following feedback from Osteoarthritis Research International members on the draft guidelines and six Delphi rounds consensus, acupuncture has been recommended for knee osteoarthritis as a non-pharmacological method [14]. Our team hopes to add clinical evidence by this trial and lay the foundation for subsequent trials.

Fire needle therapy is a special type of acupuncture, which uses specially heated and burned-red needles inserted into the acupoints or affected body region to improve symptoms. Combining heat and acupuncture, it can improve blood circulation with the help of thermal stimulations, promote the reduction of chronic inflammation, and enhance tissue regeneration [15], effectively relieving tissue edema and muscle spasm. Many doctors and therapists in China often use fire needles to treat knee osteoarthritis and find that it is effective.

Among all types of needles, the filiform needle acupuncture is commonly used in acupuncture therapy. There are some randomized controlled trials comparing the efficacy of filiform needle acupuncture and fire needle acupuncture for knee osteoarthritis. However, these studies lack scientific rigor and had a small sample size, non-standardized controls, and unfavorable outcomes.

Therefore, we designed a randomized controlled trial to evaluate the efficacy and safety of fire needle acupuncture compared to filiform needle acupuncture in knee osteoarthritis patients.

## Methods

### Study design

**Fig. 1.**
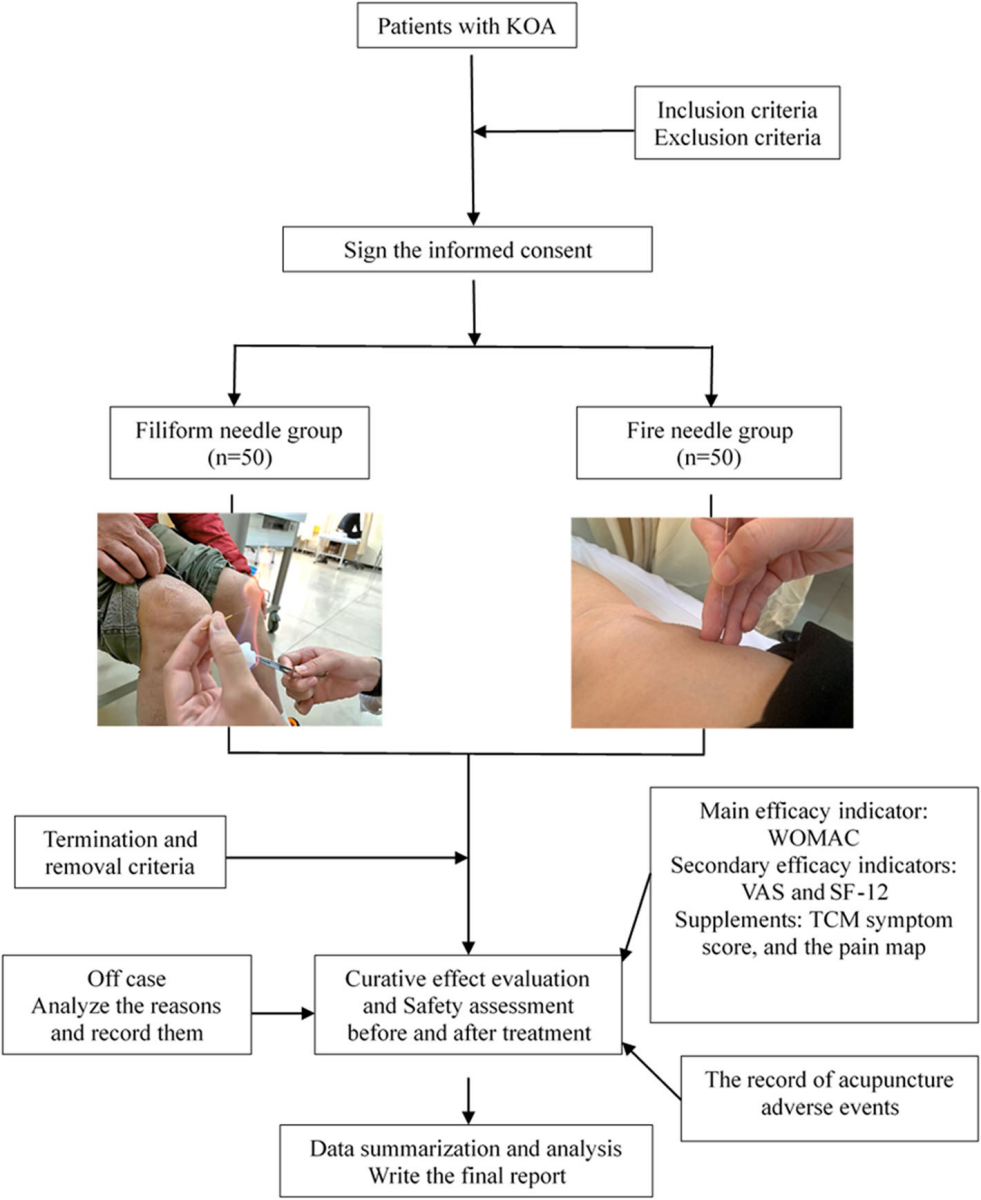
Work flow

This is a prospective randomized controlled superiority clinical trial to evaluate the efficacy and safety of fire needle acupuncture compared to filiform needle acupuncture for knee osteoarthritis. One hundred participants with knee osteoarthritis will be randomly allocated to the fire needle and filiform groups at a 1:1 ratio. The schedule of enrollment, interventions, and assessments is summarized in Table 1, and the flow diagram of the study procedure is presented in Fig. 1. Patient recruiting is from November 2020. The study protocol has been reported in accordance with the Standard Protocol Items: Recommendations for Clinical Interventional Trials (SPIRIT) guidelines (Additional file 1).

**Table 1.**
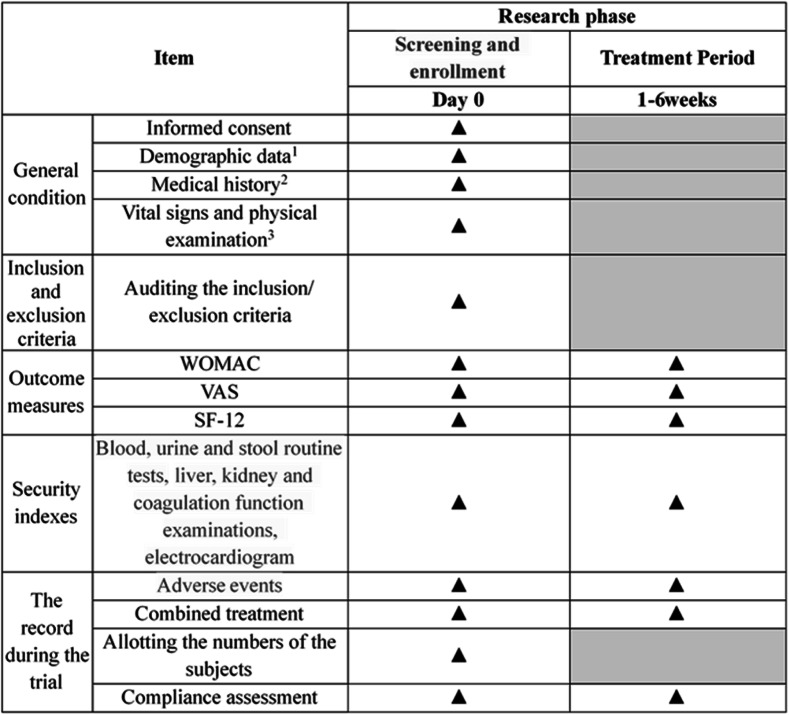
Clinical study schedule

^1^The demographic data includes age, sex, height, weight, ethnicity, marital status, and occupation

^2^The medical history includes the course of the disease, past history, the history of present illness, the history of allergy, concomitant diseases, and medication

^3^The patients’ sitting vital signs will be measured after 10 min of quiet rest

### Recruitment

Trial participants with knee osteoarthritis will be recruited from outpatient clinics at the Beijing Hospital of Traditional Chinese Medicine, Capital Medical University. Meanwhile, information flyers introducing the details of the trial will be posted at the outpatient clinics for greater exposure.

### Study procedure

This study consists of two phases including (i) a baseline phase (week 0) and (ii) a 6-week treatment phase (week 1 to week 6).

Once potential participants show interest in this trial, they will be invited to attend an eligibility assessment in which their eligibility will be assessed by trial investigators; thereafter, eligible participants will be enrolled and randomly assigned to either the fire needle group or the filiform needle group and given a 6-week treatment.

### Participants

#### Inclusion criteria

Participants who meet all of the following requirements will be considered for inclusion: (1) diagnosed as having osteoarthritis according to *American College of Rheumatology Clinical Classification Criteria for Osteoarthritis of the knee*, (2) graded II–III of Kellgren-Lawrence Radiographic Classification [16], (3) aged between 30 and 75 years old, (4) VAS score of knee joint pain is over 4 out of 10, (5) received no medication or relevant treatment in the past 2 weeks, and (6) provided written informed consent.

#### Exclusion criteria

Patients will be excluded if they have (1) non-primary knee osteoarthritis (e.g., secondary knee osteoarthritis, inflammatory, or other rheumatic diseases); (2) history of trauma, surgery, meniscus or ligament damage, or severe joint deformity on knee joint; (3) serious cardiovascular, cerebrovascular, lung, liver, spleen, kidney or hematopoietic system diseases, tumors, hemorrhagic diseases, or mental diseases; (4) difficulty in cooperating with the examination and receiving quantitative evaluation; (5) female participants who are pregnant or lactating; and (6) cicatricial or hypersensitive to acupuncture treatment.

### Randomization and allocation concealment

The randomization will be performed by the Research Centre of Clinical Epidemiology, Peking University Third Hospital. A block randomization method will be used to generate the random allocation sequence; predetermined computer-generalized randomization opaque sealed envelopes will be used to ensure the allocation concealment. The opaque sealed envelopes, with the participant’s screening order printed outside and randomly assigned group printed inside, will be numbered consecutively and connected into a strain. Researchers will enroll the eligible participants after screening, then separate and open each envelope from the strain in the sequence corresponding to the participant’s screening order and assign the eligible participant into either the fire needle group or filiform needle group.

The statistician who generates the randomly assigned sequence is not the same person as the researcher who decides whether the subject is qualified. People who generate or save the randomly assigned sequence cannot participate in the trial process.

### Blinding

Outcome assessors and personnel involved in data collection and data analysis will be blinded to participants’ group allocation throughout the entire trial. The acupuncturist and participants cannot be blinded due to the nature of the intervention, but they will be informed not to communicate with outcome assessors regarding treatment procedures and responses.

### Intervention

All participants will receive the allocated intervention twice a week for 6 weeks. All participants will go through a standardized interview and be provided with details of the study. The acupuncturist who perform treatments for both groups are registered with the Ministry of Health of the People’s Republic of China as Chinese medicine practitioners and have more than 20 years of clinical experience. Before the trial begins, all acupuncturists will receive special training regarding the purpose and standard procedure of the trial, treatment strategies, and quality control. The treatment details will be fully documented in accordance with the Standards for Reporting Interventions in Controlled Trials of Acupuncture (STRICTA) [17] and Good Clinical Practice guidelines.

#### Fire needle group

**Fig. 2.**
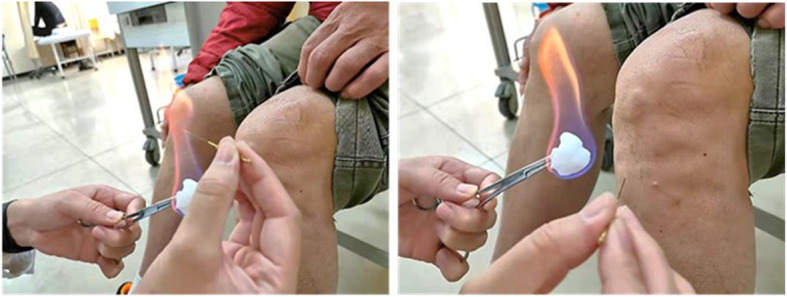
Fire needle

Acupoints including the ashi point (local pain point), bilateral ST34 (Liangqiu), bilateral SP10 (Xuehai), bilateral ST35 (Dubi), bilateral EX-LE4 (Neixiyan), and bilateral GB34 (Yanglingquan) will be acupunctured, using a tungsten manganese alloy fine fire needle with a size of 0.5× 25 mm. All locations of the acupoints will be identified according to *The Location of Acupoints*—the national standard of the People’s Republic of China (GB12346-90). Operating method (Fig. 2): participants will be treated in the supine position with the knee bent. First, the acupoints will be identified by nail scratches and then sterilized. Then the needle will be rapidly stabbed into place and pulled out, about 0.5 cm deep, three times at each point, when the tip and the middle parts of the needle body have been burned red by an alcohol lamp. After pulling out the needle, pressure will be briefly applied to the needle hole with a sterilized cotton ball to avoid bleeding, and the patients will be asked to keep these areas clean to avoid infection.

#### Filiform needle group

**Fig. 3.**
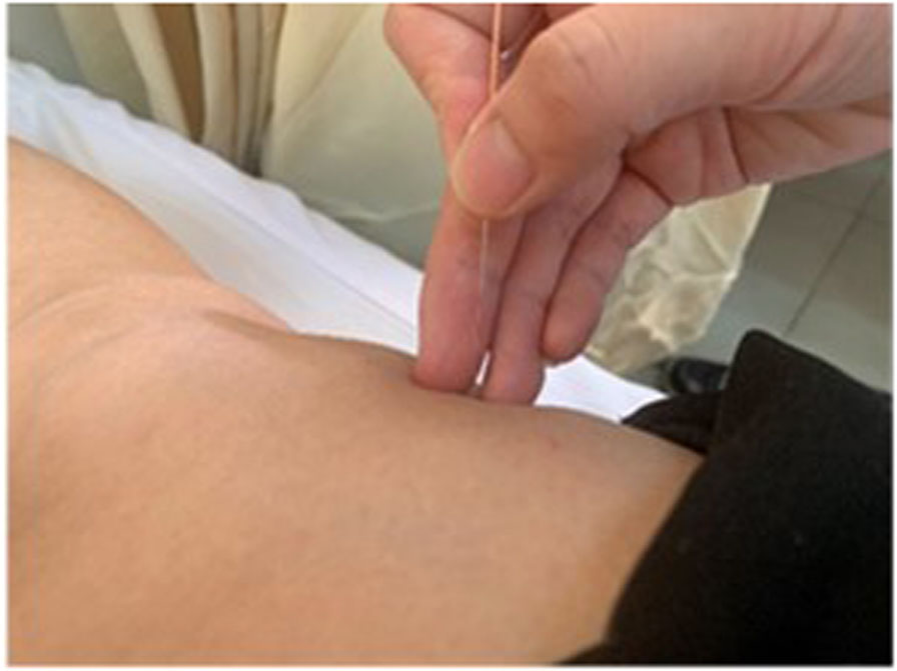
Filiform needle

The same acupoints will be selected for acupuncture treatment by the filiform needle (the name of the brand is Andi), with a size of 0.3 × 40 mm. According to the patient’s somatotype, vertically stick the needle in 15–25 mm at the points. Operating method (Fig. 3) is mild reinforcing-attenuating, evenly lifting, inserting, and twisting, then retaining the needle for 20 min.

### Outcome measures

The efficacy of the treated patients will be evaluated before treatment and 6 weeks after intervention. The main efficacy indicator is the WOMAC questionnaire for knee pain, stiffness, and function. The secondary efficacy outcomes are VAS for generalized pain and SF-12 for quality of life. The collected information and data will be maintained confidentially.

#### WOMAC (Western Ontario and McMaster Universities Osteoarthritis Index)

WOMAC is a standardized questionnaire used to evaluate the condition of subjects with osteoarthritis of the knee and hip, which assesses the three dimensions of pain, stiffness, and functional limitation [18] with 24 questions. The score range for each question is 0 to 4. The total score of WOMAC is on a scale of 0 to 96, and higher scores indicate more severe symptoms.

#### VAS (visual analog scale)

The VAS score is used to assess knee joint pain. It ranges from 0 to 10, and a higher score means more severe pain. The efficacy evaluation mainly refers to the Outcome Measures in Rheumatological Clinical Trials formulated by the Osteoarthritis Research Society International (OMERACT-OARSI), i.e., the pain has been highly improved when the score decreases ≥ 50% and the absolute value declines ≥ 2; pain has been improved to some extent when the score decreases ≥ 20% and the absolute value declines ≥ 1, which is the minimum variation for clinical significance.

#### SF-12 (12-item Short Form Health Survey)

**Fig. 4.**
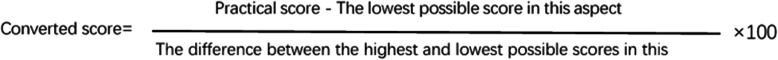
Conversion formula

The SF-12 scale consists of two parts: physical component subscale and mental component subscale. Converted by a specific formula, the total score is on a scale of 0 to 800, and higher scores represent better health. The conversion formula is presented in Fig. 4. The conversion formula is presented in Fig. 4.

### Safety assessments

Adverse reactions related to acupuncture include pain, fainting, bleeding, hematoma, infection, nerve injury, aggravation of an underlying disease, severe skin allergy, stuck needle (i.e., the therapist feels sluggish and astringent under the needle and has difficulty twisting, lifting, and inserting the needle, and the patient feels pain during the acupuncture process [19]), a bent needle, and a broken needle. The classification of adverse events refers to adverse drug reactions: level 1—safe, without any adverse reactions; level 2—relatively safe. If there are adverse reactions, the participants can be treated sequentially without any treatment of the complication; level 3—there are safety problems and moderate adverse reactions. The participants can continue to receive treatment after the reactions have been taken care; and level 4—the treatment was terminated due to the adverse reaction. Constitutional or local symptoms, signs, and adverse reactions will be dynamically observed and recorded, which will be used for comprehensive analysis.

For adverse reactions related to the trial, the therapists will give symptomatic treatment such as hemostasis, disinfection, and anti-inflammatory according to the specific situation.

### Data management

The study will be regularly monitored by the Data Management and Monitoring Committee of the Good Clinical Practice Department of Beijing Hospital of Traditional Chinese Medicine, Capital Medical University. The Data Management and Monitoring Committee is independent of the trial researchers and has no competing interests. The Data Management and Monitoring Committee will monitor the overall quality and integrity of the data, examine the original case report forms, interview the researchers, verify the record of adverse events, and confirm that the study conforms to the principles of this protocol.

### Sample size calculation

In a previous study [20], the WOMAC score after treating by fire needle was 52.83 ± 20.85 and 59.92 ± 23.80 days in the filiform needle group. Based on the relevant literature [21] we have consulted, the minimal clinically important difference (MCID) of WOMAC scores was set at 6.7. It is set that the inspection level is 0.05 (*α* = 0.05, two-sided), and the power of the test is 0.2 (*β* = 0.2), using a 1:1 ratio, i.e., the sample size of fire needle group (*n*_1_) = the one of filiform needle group = 40. Considering the loss rate of 20% or less, a sample size of 50 per group and a total number of 100 participants will be recruited during the entire study accordingly.

### Statistical analysis

Using the statistical software of SPSS 20.0, *p* < 0.05 for a two-sided test will be regarded as the standard to judge the significance of the difference. The measurement data will be expressed as mean ± standard deviation ($\overline{x}$ ± *s*). If the precondition of a parametric test is satisfied, *t* test will be chosen. For non-parametric data, a Wilcoxon rank sum test will be used. The counting data will be expressed as frequency (or rate), which is calculated by a chi-square test or Fisher’s exact test.

The results of the study will be statistically analyzed according to the principle of intention-to-treat (ITT) and per-protocol population (PP). In ITT, all enrolled participants who have treated at least once will be analyzed. In PP, all participants who meet the study criteria, have completed the observation, have good compliance, do not use prohibited drugs during the study, and completed the study contents will be analyzed. Finally, comparison of the consistency of the results between ITT and PP will be conducted. The multiple imputation method will be used to process the missing data for the primary outcome.

### Ethics and dissemination

This study will adhere to the principles of the *Declaration of Helsinki (2008)*, *Measures for the Ethical Review of Biomedical Research Involving Humans (2007)* issued by the Ministry of Health, and *Administrative Regulations on the Ethical Review of TCM clinical research (2010)* issued by State Administration of Traditional Chinese Medicine of the People’s Republic of China, and it has been approved by the Research Ethics Committee of Beijing Hospital of Traditional Chinese Medicine, Capital Medical University, on 8 June 2018 (reference 2018SB-066). The study has been registered with the Chinese Clinical Trial Registry (ChiCTR) on 18 November 2018, and the registration number is ChiCTR1800019579. The results will be disseminated through publications in open source scientific peer-reviewed journals, presentations in scientific conferences, or a master’s thesis. The identifying images or other personal or clinical details of participants that compromise anonymity will not be published.

## Discussion

As a chronic degenerative disease, osteoarthritis involves younger patients with a high incidence and substantial long-term disability rate. Joint pain and limitation of motion in osteoarthritis subjects lead to decreased muscle strength, changes in the biomechanical axis, and disuse atrophy of the muscle, resulting in changes in the function of the lower limbs, changes in joint stress distribution, and decreased joint stability, entering a vicious cycle [22]. At present, there are limitations of medical and other treatment methods [3], which makes the advantages of acupuncture more prominent. This study will evaluate the efficacy and safety of fire needle and ordinary filiform needle acupuncture for knee osteoarthritis by clinically comparative observation, which lays a foundation for subsequent large-scale multi-center randomized controlled trials. It may also contribute to the optimization of the clinical treatment plan for knee osteoarthritis with fire needle and the internationalization of fire needle therapy.

Acupuncture is popular in China due to its long history, simple operation, good efficacy, low cost, relative safety, and other advantages, and has been demanded as a complementary and alternative medicine by more and more subjects all over the world. As a special kind of acupuncture, it is believed in TCM to spell out that fire needle acupuncture has the dual effects of dredging the meridian through stabbing and dissipating cold through the heat, which can enhance the circulation of blood and qi. Osteoarthritis is one of the diseases recommended by the World Health Organization to be treated with acupuncture and moxibustion [23]. Studies have shown that broil stimulation can dilate the blood vessels in the affected parts, improve the osmotic pressure, stimulate the stress response of the body, and promote self-repair ability [24]; inhibit the local immune response and eliminate joint swelling and stiffness to relieve pain [25]; promote the formation of benign self-regulation mechanism, cell growth factors including vascular endothelial growth factor (VEGF), promote the growth of granulation tissue, and accelerate the closure of wounds [26], so as to relax the muscles and tendons, nourish the joints, and relieve pain. The selection of acupoints is based on the acupuncture theory of TCM, decades of clinical experience in our department, and systematic reviews [27, 28].

This study will compare the clinical efficacy for treating knee osteoarthritis between fire needle and filiform needle acupuncture, so the subjects will be randomly allocated to the fire needle group or the filiform needle group. It is difficult to have a placebo control group with no acupuncture treatment, since the subjects who come to the acupuncture and moxibustion departments are all expecting acupuncture treatment. At present, there are generally two methods for placebo control of sham needle or acupuncturing non-acupoint points which are not related to the treatment or not inserting into the subcutaneous area. However, both two methods are questionable and remain to be discussed [3, 29, 30], so the sham needle group will not be set up in this study.

This study is a prospective randomized controlled trial, using randomization and allocation concealment to decrease selection bias and using blinding method to reduce information bias. However, it is difficult to be double-blinded in practice because both doctors and subjects can see and feel the difference between fire needle acupuncture and filiform needle acupuncture, especially Chinese people, who are particularly familiar with acupuncture and moxibustion. Therefore, only the third-party evaluator will be blinded in this study.

### Trial status

Recruitment began in November 2020 and will end in November 2021.

This protocol is the first version.

Date: January 8, 2020

## Supplementary Information

**Additional file 1.** : SPIRIT 2013 Checklist

## Data Availability

We will share the data after the trial is finished. The full data set will be available by an author contact when this trial is completed and published.

## Abbreviations

TCM: Traditional Chinese medicine
WOMAC: Western Ontario and McMaster Universities Osteoarthritis Index
VAS: Visual analog scale
SF-12: 12-item Short Form Health Survey
ITT: Intention-to-treat
PP: Per-protocol population
